# A Novel Prototype Biosensor Array Electrode System for Detecting the Bacterial Pathogen *Salmonella typhimurium*

**DOI:** 10.3390/bios12060389

**Published:** 2022-06-04

**Authors:** Palaniappan Ramasamy, Gajalakshmi Dakshinamoorthy, Shanmugam Jayashree, Dhamodharan Prabhu, Sundararaj Rajamanikandan, Palaniyandi Velusamy, Govindan Dayanithi, Robert E. B. Hanna

**Affiliations:** 1 Research and Development Wing, Bharath Institute of Higher Education and Research (BIHER), Sree Balaji Medical College and Hospital (SBMCH), Chromepet, Chennai 600044, Tamil Nadu, India; prabhu_research@sbmch.ac.in (D.P.); rajamanikandan_research@sbmch.ac.in (S.R.); velusamy.research@bharathuniv.ac.in (P.V.); govindan.dayanithi@umontpellier.fr (G.D.); 2 Department of Biotechnology, University of Madras, Chennai 600025, Tamil Nadu, India; gdakshinamoorthy@guardanthealth.com (G.D.); jayashree@stellamariscollege.edu.in (S.J.); 3 MRD Tech Development, 505 Penobcot Dr., Redwood City, CA 94063, USA; 4 Department of Biotechnology, Stella Maris College, Chennai 600086, Tamil Nadu, India; 5 Molecular Mechanisms in Neurodegenerative Diseases Laboratory (MMDN), University of Montpellier, L’École Pratique des Hautes Etudes-Sorbonne, INSERM, UMR-S1198, CEDEX 5, 34095 Montpellier, France; 6 School of Biology and Biochemistry, The Queen’s University of Belfast, Belfast BT7 1NN, UK; bob.hanna@afbini.gov.uk; 7 Veterinary Science Division, Agri-Food and Biosciences Institute, Belfast BT4 3SD, UK

**Keywords:** *Salmonella* species, biosensor, immunosensor, dialysis membrane electrode interface disc, glassy carbon electrode, salmonellosis

## Abstract

Salmonellosis caused by *Salmonella* sp. has long been reported all over the world. Despite the availability of various diagnostic methods, easy and effective detection systems are still required. This report describes a dialysis membrane electrode interface disc with immobilized specific antibodies to capture antigenic *Salmonella* cells. The interaction of a specific *Salmonella* antigen with a mouse anti-*Salmonella* monoclonal antibody complexed to rabbit anti-mouse secondary antibody conjugated with HRP and the substrate o-aminophenol resulted in a response signal output current measured using two electrode systems (cadmium reference electrode and glassy carbon working electrode) and an agilent HP34401A 6.5 digital multimeter without a potentiostat or applied potential input. A maximum response signal output current was recorded for various concentrations of *Salmonella* viz., 3, 30, 300, 3000, 30,000 and 300,000 cells. The biosensor has a detection limit of three cells, which is very sensitive when compared with other detection sensors. Little non-specific response was observed using *Streptococcus*, *Vibrio*, and *Pseudomonas* sp. The maximum response signal output current for a dialysis membrane electrode interface disc was greater than that for gelatin, collagen, and agarose. The device and technique have a range of biological applications. This novel detection system has great potential for future development and application in surveillance for microbial pathogens.

## 1. Introduction

Salmonellosis continues to plague human populations in both developed and developing countries. According to the World Health Organization, salmonellosis is projected to affect over 550 million people worldwide including 220 million people under the age of 5 years [1,2]. *Salmonella* is one of the major foodborne pathogens and all the species of *Salmonella* are known to be pathogenic, causing morbidity and mortality in both humans and animals [3]. Within the genus, *Salmonella typhimurium* causes gastroenteritis leading to diarrhea, abdominal cramps, vomiting, and fever, while *Salmonella typhi* causes typhoid fever, leading to complications including liver damage, swelling of the heart and gut and internal bleeding [4]. Early detection, diagnosis and treatment of *Salmonella* infections is important to control the spread of infection [5]. At present, disease control and prevention relies upon the basic diagnostic methods that are currently used in clinical medicine, food safety and environmental settings. Various conventional methods exist for the detection and identification of *Salmonella* sp., largely dependent on standard culture techniques involving the use of enrichment and selective media, as well as specific tests for the ability of the organism to grow under a range of environmental conditions [6].

Biochemical and serological tests are widely used for detection of *Salmonella* sp. [7]. Several techniques, viz. flow cytometry, optical and calorimetry methods, ultrasound techniques, radiometry, infrared (IR) spectroscopy, and microbial identification systems have also been used to identify *Salmonella* sp., though they are prohibitively labor-intensive and time-consuming, requiring a week to obtain reliable results [6]. In addition, they are inappropriate for testing a large number of samples [7,8,9,10,11,12,13,14,15]. Some newer technologies such as polymerase chain reaction (PCR) and enzyme linked immunosorbent assay (ELISA) are very sensitive but analysis time is protracted [10,11,12]. A number of other tools are available for the diagnosis of a wide range of pathogenic bacteria including: electrochemical immunosensors, genosensors, aptasensors and phagosensors [13,16,17,18,19,20], nanoparticle-based bio-barcoded DNA sensor [11,12,21,22], electrochemical DNA biosensor consisting of nanoporous glassy carbon electrode with differential pulse voltammetry (DPV) and electrochemical impedance spectroscopy (EIS) [23,24,25] microfluidic nano-biosensor, *Salmonella* aptasensor, impedimetric potentiometric magnetic immunoassay, label-free impedimetric biosensor and amperometric immunoassays [26,27,28,29,30,31,32,33,34,35,36,37]. MALDI-TOF has limited ability to distinguish between closely related species, which may be due to the organism’s inherent similarities [38]. Despite the fact that smartphone-based sensors for detecting pathogens have been developed, it is still unclear if they have sufficient sensitivity to discriminate between species. Limited resolution, and variance across devices are also problematic features [39,40].

The proposed technique is sensitive, specific, rapid, accurate, does not require labeling, and is cost-effective. In the current study, the aim was to develop a electrochemical-based prototype device for the detection of the foodborne pathogen, *Salmonella typhimurium*. The prototype was constructed by immobilizing *Salmonella* monoclonal antibodies on a glassy carbon biomembrane electrode interface disc to capture the specific enzyme-substrate reaction through measurement of the response signal output current. Change in impedance occurred after selective capturing of the target antigen by the specific *Salmonella* monoclonal antibody on the surface of the electrodes, and was evaluated using Agilent software. A thorough study has been performed on the immobilization of antibodies with different membranes, using various concentrations of antibodies and antigens. Additionally, the sensor’s sensitivity and specificity were tested using bacterial genera other than *Salmonella*. 

Electrochemical biosensors are based on enzymatic catalysis of a substrate producing or consuming electrons, and a variety of devices are used to measure the output voltage. The amperometric method consists of three electrodes, whereas potentiometic methods include biological and chemical field-effect transistor (FET) sensors, miniature FETs and ion-sensitive FETs [41]. However, most of these tools and methods are complex and laborious, requiring a higher cost and longer time for detection, with limitations on sensitivity. Thus, there exists a need to develop simple tools and techniques for easy use and rapid detection of *Salmonella* sp. at a low concentration of cells. In the current study, a prototype device and method consisting of antigenic *Salmonella* cells immobilized on a biomembrane electrode interface disc (dialysis membrane electrode interface disc, collagen, gelatin or agarose) was found to produce a measurable response signal output current through specific enzyme-substrate reactions. The response signal output current generated using a two-electrode system was measured with the Agilent HP34401A 6.5 digital multimeter. 

The aims of the study were: (i) to develop a new approach for detecting antigen-antibody interactions using an enzyme-substrate chemical reaction that has better detection limits than previous sensors; (ii) to create an electrochemical-based prototype device for the detection of *Salmonella typhimurium*, a foodborne pathogen, by immobilizing *Salmonella* monoclonal antibodies on a glassy carbon electrode interfaced with a disposable biomembrane electrode interface disc that enables capture of the specific enzyme-substrate reaction via a response signal output current; (iii) to choose from agarose, gelatin, collagen, and dialysis membrane, the best biomembrane electrode interface disc for electrode fabrication and primary *Salmonella* monoclonal antibody immobilization, based on the maximum response signal output current generated; (iv) to record the response signal output current curve from the fabricated glassy carbon electrode carrying a disposable dialysis membrane interface disc following the addition of, respectively, phosphate buffer, mouse anti-*Salmonella* monoclonal antibody immobilized on the membrane, bovine serum albumin, *Salmonella* cells, rabbit anti-mouse secondary antibody-enzyme conjugate, o-aminophenol substrate; and (v) finally to determine the optimal concentration of specific *Salmonella* antigen cells, the lowest detection limit of *Salmonella* cells, the range of response signal output current produced by varying *Salmonella* cell concentrations (antigen), and the relationship between detected response signal output current and *Salmonella* cell concentrations.

## 2. Methods

### 2.1. Equipment and Chemicals

#### 2.1.1. Measuring Equipment—Hewlett Packard-Agilent, HP34401A 6.5 Digital Multimeter

The measuring equipment used for monitoring and detecting *S. typhimurium* based on the response signal output current from the biosensor electrode was the Hewlett Packard-Agilent, HP34401A 6.5 digital multimeter (Agilent Technologies, Inc., Santa Clara, CA, USA). This was used to measure all electrochemical outputs, in particular the magnitude of the response signal output current. The Agilent software was used to visualize and interpret the detected signal. Electrodes have been fabricated from platinum, gold, carbon (i.e., graphite) and silicon compounds, depending on the analyte. They are known to be chemically stable and conductive. In the present study, a conventional electrode system consisting of glassy carbon as the working electrode and cadmium as the reference electrode were used. For comparison, silver and copper electrodes were also assessed as reference electrodes. All the experiments were performed at room temperature (25 °C). Using this Agilent HP34401A equipment, experiments were conducted to study the responses of the electrodes and their characteristics such as accuracy, response time, and reproducibility.

#### 2.1.2. Chemicals

Anti-*Salmonella* antibody (ICN 5974b) raised against *Salmonella* serotype E purified in mouse serum was purchased from ICN. Inc. USA. Rabbit anti-mouse secondary antibody conjugated with horseradish peroxidase (HRP) (EC1.11.1.7) was obtained from Sigma, USA. Dialysis membrane 110 with 12–14 kDa pore size was purchased from Hi-media, India. Agarose was provided by AB Gene, India. Collagen membrane was provided by Cologenesis HealthCare Pvt Ltd., Salem, India. Bind silane A-174 PLUS ONE was obtained from Pharmacia Biotech, Uppsala, Sweden. Glutaraldehyde (4% *v*/*v* solution in double distilled deionized water) was obtained from Agar Scientific Limited, Essex, UK. Bovine Serum Albumin (BSA) (1% *v*/*v* solution in double distilled deionized water) was obtained from the Sisco Research Laboratories Pvt. Ltd., Mumbai, Maharashtra, India. Ortho-amino phenol and gelatin were supplied by LOBA Chemie, India. Hydrogen peroxide (1% *v*/*v* solution in double distilled deionized water) was obtained from Central Drugs and Pharmaceutical, Chennai, India. Potassium phosphate (KH_2_PO_4_), di-potassium hydrogen orthophosphate (K_2_HPO_4_), sodium chloride (NaCI), acetone and formaldehyde (37–41% *w*/*v* solution) were obtained from Ranbaxy Fine Chemicals Ltd., New Delhi, India. All the chemicals used were of analytical grade and the solutions were prepared with double distilled deionized water.

#### 2.1.3. Buffer Solutions

The effects of buffer concentration and pH on the sensitivity of antibodies immobilized on the sensor surface were evaluated [42]. The optimum pH and concentration of phosphate buffered saline for maximum sensitivity were found to be 7.2 and 0.1 M, respectively. Yao and Zhou, 1988 [43], reported that the immobilized antibody and *Salmonella* antigen interact with PBS ions in a complex manner. Hence, in the current study, experiments were carried out making use of 0.1 M PBS (pH 7.2) as a reaction buffer. Phosphate buffer stock solution (0.2 M) was prepared by mixing solutions containing 0.2 M KH_2_PO_4_ and K_2_HPO_4_ to a pH of 7.2.

#### 2.1.4. Formulation of Specific Anti-*Salmonella* Antibody

Serial dilutions of mouse anti-*Salmonella* monoclonal antibody stock solution were prepared using 0.1 M PBS pH 7.2 containing 0.9% NaCl. The dilution factors for antibody stock solutions were as follows: 1:1000, 1:10,000, 1:20,000, 1:30,000, 1:40,000, 1:50,000 and 1:60,000.

#### 2.1.5. Formulation of HRP Conjugated Secondary Antibody

One µL of rabbit anti-mouse secondary antibody conjugated with HRP was taken from the stock and made up to 1 mL using 0.1 M PBS pH 7.2.

#### 2.1.6. Formulation of Substrate

An appropriate amount of o-amino phenol (2 mg) with 1.4% of hydrogen peroxide was dissolved in 1 mL of 0.1 M PBS pH 7.2.

### 2.2. Preparation of *Salmonella* Antigen and Source of Cultures

*S. typhimurium* (*synonyms*: *Salmonella typhimurium*; *S. enterica* serovar Typhimurium) *stock* culture from the American type culture collection (ATCC 23564), and *Pseudomonas* sp., (ATCC25619), *Vibrio* sp., (ATCC17802) and *Streptococcus* sp. from our own cultures, were used in these studies. The identity of cultures was confirmed using traditional biochemical, cell morphology and serologic tests. The stock cultures of *Salmonella* sp., *Pseudomonas* sp., and *Vibrio* sp., were maintained in nutrient broth, whereas for *Streptococcus* sp., trypticase soybean broth was used.

### 2.3. Growth of Cultures

*S. typhimurium* and *Pseudomonas* sp., cultures were streaked for isolation on nutrient agar plates. For *Vibrio* sp., and *Streptococcus* sp., TCBS and TSA culture plates were used, respectively, before inoculation of the fresh culture in broth for overnight incubation at 37 °C in a shaking incubator. 

### 2.4. Determination of Cell Concentration

The cells of *Salmonella* sp., *Vibrio* sp., *Pseudomonas* sp. and *Streptococcus* sp., were serially diluted with 0.1 M PBS to 1:10^5^, 1:10^4^, 1:10^3^ and 1:10^2^. The number of viable cells in each dilution was determined by spread plating 0.1 mL of each dilution onto duplicate plates of nutrient agar, TCBS and TSA separately for each culture, and incubating for 24 h at 37 °C before making a final count of CFU/mL and calculating the average CFU. The tubes with diluted cells were analyzed spectrophotometrically at the absorbance of 600 nm and the concentration of the cells was estimated.

### 2.5. Electrode Preparation

#### 2.5.1. Method for Antigen Preparation

The cells of *Salmonella* sp., *Vibrio* sp., *Pseudomonas* sp. and *Streptococcus* sp., from the broth were fixed separately by treating with 10% formaldehyde for 30 min at 10 °C. The fixed cells were then washed by centrifugation (13,416× *g* for 10 min) and resuspended in 10 mL of sterile 0.1 M PBS (pH 7.2), then centrifuged again and resuspended in 500 µL of PBS. The concentrated cells were then heat-treated in a boiling water bath at 100 °C for 10 min and used for the experiments.

#### 2.5.2. Electrode Fabrication

**Figure 1:**
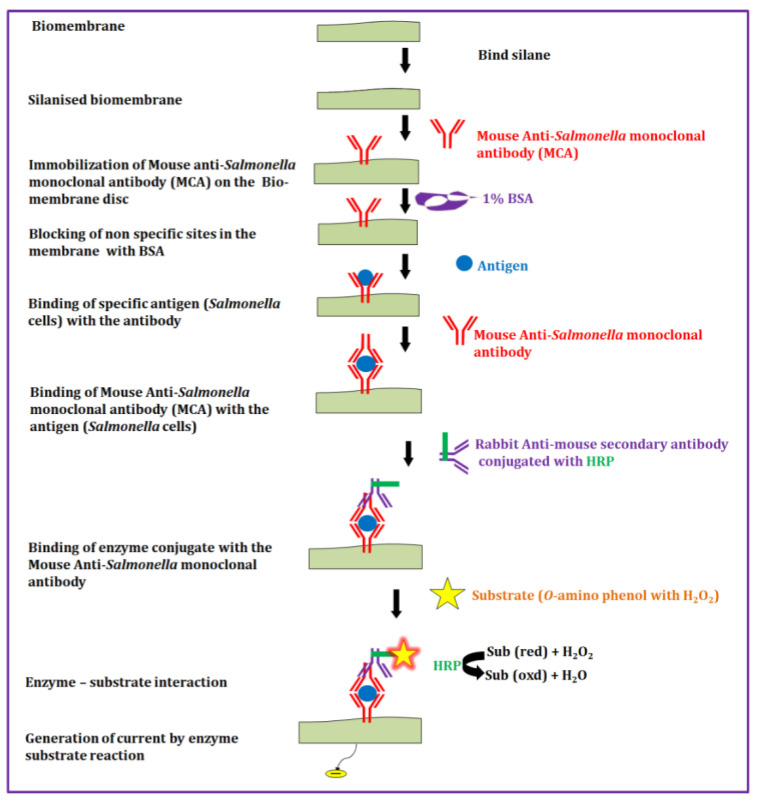
Fabrication of biosensor biomembrane electrode interface disc for *Salmonella* detection.

Copper wire was inserted at one end of a 1 cm long cylindrical rod of glassy carbon with a diameter of 0.6 cm (R = 3 mm; area = 28.27 mm^2^, circumference = 18.84 mm) to make electrodes. Before each experiment, the other end of the glassy carbon rod was polished on fine emery paper and thoroughly washed with acetone and 0.1 M PBS solution. After drying, the free end of the electrode surface was overlaid with a disposable biomembrane electrode interface discs (collagen, agarose, gelatin or dialysis tubing). The biomembrane electrode interface disc was secured to the electrode with an O-ring, and the surface of the membrane was coated with 1 µL of binding silane which was allowed to vaporize in a dust-free environment (Figure 1).

#### 2.5.3. Immobilization of Monoclonal Antibodies

The silane-treated biomembrane electrode interface disc was coated with a layer of mouse anti-*Salmonella* monoclonal antibody *(Salmonella* serogroup E) that was anticipated to capture the specific pathogen *Salmonella*. The coating procedure was as follows: the sensor surface was incubated for 15 min with 600 µL of monoclonal antibody to *Salmonella* serogroup E (1:1000 dilution). The sensor surface was then bathed three times with PBS. Finally, all non-specific binding sites were blocked by incubation for 15 min with 600 µL of 1% bovine serum albumin (BSA) solution in PBS. Excess bovine serum was removed by washing with PBS.

In this study, an innovative approach using the primary mouse anti-*Salmonella* monoclonal antibody twice was proved to enhance the binding of *Salmonella* antigen to the antibody-enzyme substrate complex and as a result, a higher electrical signal was generated and detected. By means of appropriate control reactions, this study has also verified that non-specific binding of the rabbit anti-mouse secondary antibody conjugated with HRP to antigen-bound primary mouse anti-*Salmonella* antibody was not reflected in terms of current production. Only the reaction of the o-aminophenol substrate with the secondary antibody-bound enzyme (HRP) resulted in the oxidation of the substrate to produce the quinone compound, 3-amino phenol phenoxazine. This reaction was the source to measure the response signal output current. The enzyme reaction proceeds according to the formula:

HRP
sub(red) + H_2_O_2_ → sub(oxd) + H_2_O (1)

Here, sub(red) refers to the reduced state of the substrate that is oxidized to sub(oxd) as the catalytic product. Since sub(oxd) can be regenerated, further reduction can continue to occur at the electrode surface, with continued generation of a response signal output current. Similarly, Li et al., 2002 [44] reported the generation of catalytic current using voltametric ELISA-antibody-bound enzyme (HRP), with oxidation of o-aminophenol to produce a quinone compound, 3 amino phenol phenoxazine.

#### 2.5.4. Bacteria Binding Measurements

The sensor surface covered with the disposable biomembrane electrode interface disc on which the antibody film was immobilized, was positioned in the probe arm of the instrument just before the delivery of the analyte solutions into the electrode reaction chamber. Immediately before the recording was started, 600 µL of the control solution (PBS) was delivered into the electrode reaction chamber and the response signal output current was recorded for 15 min. The control solution (PBS) was carefully removed, and a new recording of output response signal was started with the addition of 600 µL of a solution containing monoclonal antibodies *Salmonella* serogroup E, followed by immobilization of *Salmonella* cells on the electrode membrane surface using a sample of 10 µL containing 30 cells, 300 cells, 3000 cells, 30,000 cells, or 300,000 cells at dilutions of 3 × 10^3^, 3 × 10^4^, 3 × 10^5^, 3 × 10^6^, and 3 × 10^7^ *Salmonella* cells/mL, respectively. This was followed by antibody-enzyme conjugate (Rabbit anti-mouse secondary antibody conjugated with Horseradish peroxidase (HRP) (EC 1.11.1.7)) and enzyme-substrate (o-amino phenol (2 mg) with 1.4 percent hydrogen peroxide, dissolved in 1 mL of 0.1 M PBS pH 7.2), which were placed consecutively in the electrode chamber. The data collected were stored and analyzed.

### 2.6. Experiments

In this study, five different experiments were carried out: Experiment 1, selection of suitable membrane for the immobilization; Experiment 2, determination of antigen-antibody interaction via immuno enzyme substrate reaction; Experiment 3, optimization of immobilized primary monoclonal antibody concentration; Experiment 4, optimization of *Salmonella* antigen concentration, and Experiment 5, to demonstrate the specificity of the sensor.

#### 2.6.1. Experiment 1

**Figure 2:**
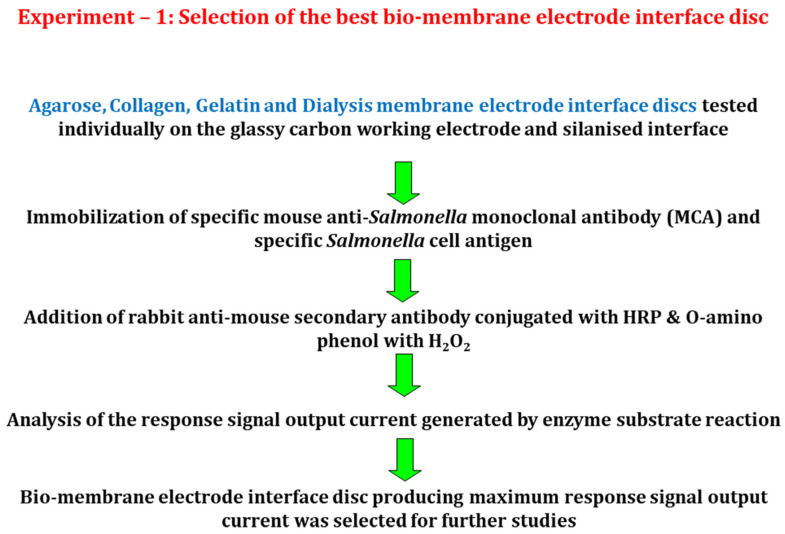
Schematic diagram showing the process of selecting the best biomembrane electrode interface disc on the basis of response signal output current.

In this experiment, a suitable disposable biomembrane electrode interface disc for the immobilization of mouse anti-*Salmonella* monoclonal antibody was selected on the basis of response signal output current output generated by each of the membranes. Membranes used for the electrode fabrication were agarose, gelatin, collagen and dialysis tubing. Each of these membranes was laid individually over the electrode surface, silanized and tested by the procedure as described above. The dilution of primary mouse anti-*Salmonella* monoclonal antibody immobilized on the sensor surface was 1:1000 and the *Salmonella* cell concentration used was 3000 cells (10 µL of 3 × 10^5^ cells/mL). The membrane that generated the maximum response signal output current was chosen as the most suitable surface for the immobilization (Figure 2).

#### 2.6.2. Experiment 2

**Figure 3:**
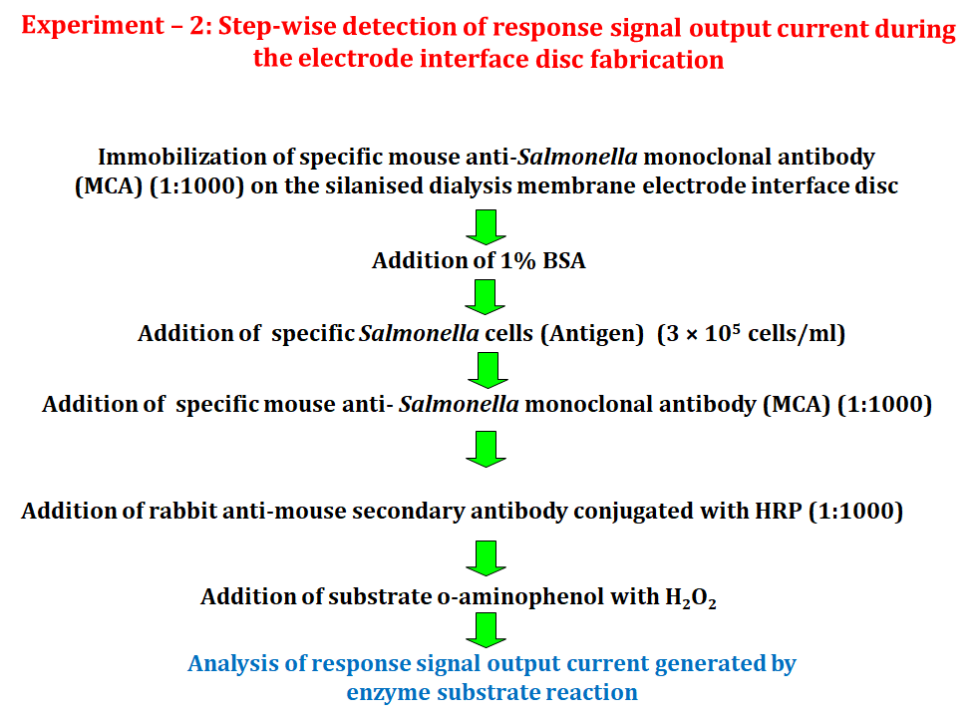
A diagram depicting the various stages involved in the *Salmonella* cell detection experiment.

In this experiment, the interaction of *Salmonella* cells antigen with mouse anti-*Salmonella* monoclonal antibody (MCA) was determined through an enzyme substrate reaction, following the procedure described in Section 2.5.4. The response signal output current generated was recorded. A dialysis membrane electrode interface disc was used in this experiment to immobilize the mouse anti-*Salmonella* monoclonal antibody (MCA) (1:1000), which was allowed to interact with *Salmonella* antigen, 3000 cells (10 µL of 3 × 10^5^ cells/mL) (Figure 3). 

#### 2.6.3. Experiment 3

**Figure 4:**
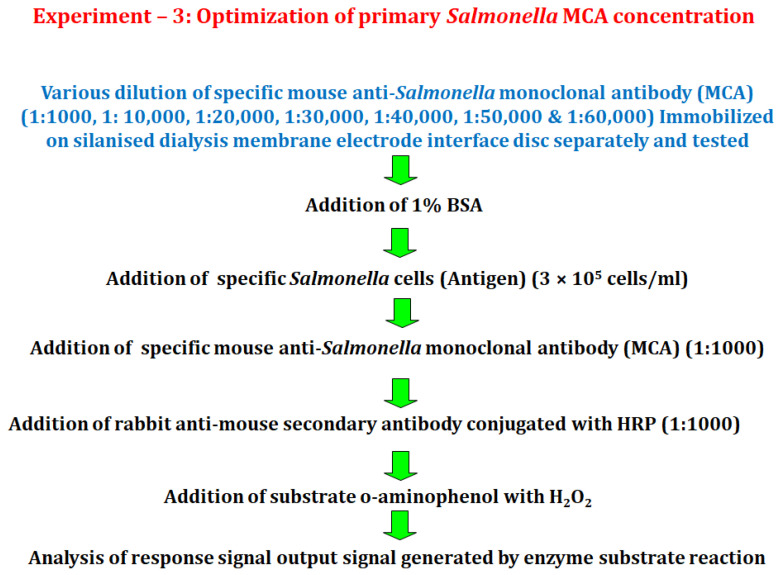
Schematic diagram showing optimization of mouse anti-*Salmonella* monoclonal antibody (MCA) concentration for the detection of *Salmonella* cells.

In this experiment, an electrode fabricated with a dialysis membrane electrode interface disc was used to study the influence on the detection signal caused by varying the concentration of immobilized mouse anti-*Salmonella* monoclonal antibody (MCA). The *Salmonella* cell (antigen) concentration used was 3000 cells (10 µL of 3 × 10^5^ cells/mL) and the dilutions of the primary mouse anti-*Salmonella* monoclonal antibody (MCA) were as follows 1:1000, 1:10,000, 1:20,000, 1:30,000, 1:40,000, 1:50,000 and 1:60,000. The rest of the steps followed for this experiment were the same as explained above and the response signal output current for each cell concentration was recorded (Figure 4).

#### 2.6.4. Experiment 4

**Figure 5:**
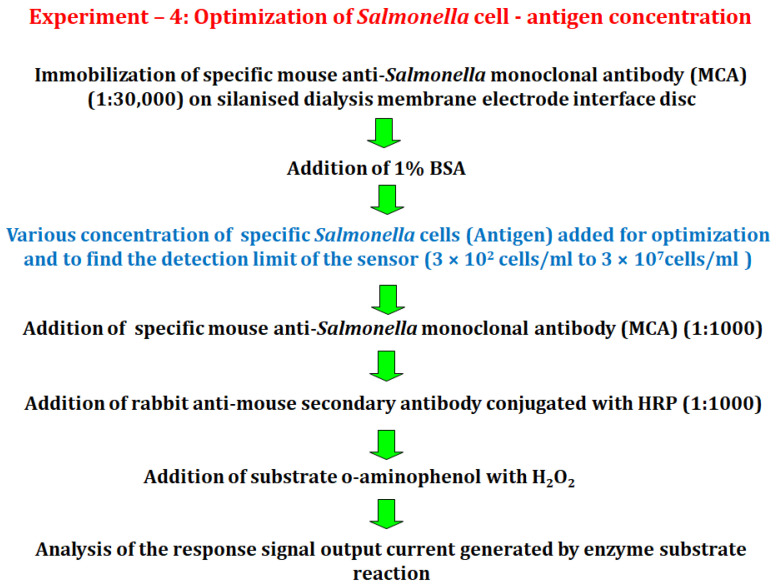
A schematic illustration depicting the optimization of *Salmonella* antigen cell concentration for *Salmonella* detection.

In this experiment, the detection limit for the electrode with attached dialysis membrane electrode interface disc was determined by varying the *Salmonella* cell concentration, with the optimized concentration of primary antibody (mouse anti-*Salmonella* monoclonal antibody (MCA)) immobilized on the membrane. The range of concentrations of *Salmonella* cells used was 300,000 cells (10 µL of 3 × 10^7^ cells/mL), 30,000 cells (10 µL of 3 × 10^6^ cells/mL), 3000 cells (10 µL of 3 × 10^5^ cells/mL), 300 cells (10 µL of 3 × 10^4^ cells/mL), 30 cells (10 µL of 3 × 10^3^ cells/mL) and 3 cells (3 × 10^2^ cells/mL) (Figure 5).

#### 2.6.5. Experiment 5

**Figure 6:**
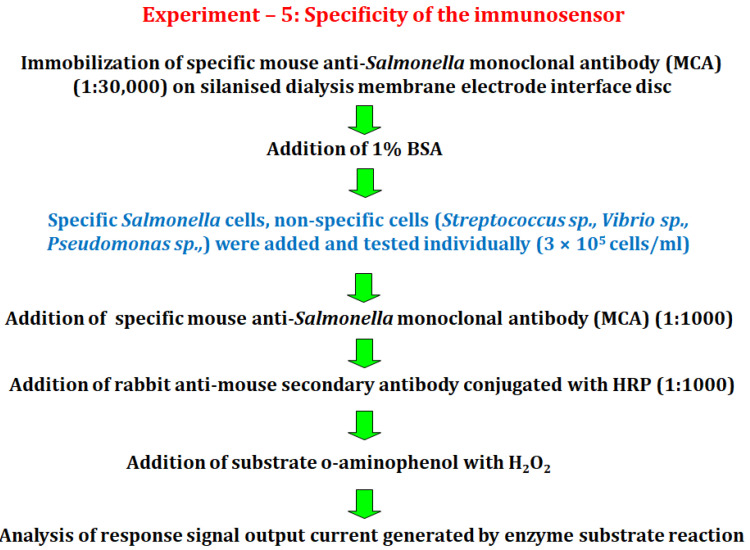
Diagram showing the processes for specific detection of *Salmonella* cells.

In this experiment, the effects of non-specific organism like *Vibrio* sp., *Pseudomonas* sp., and *Streptococcus* sp., were investigated in order to verify the specificity of the immunosensor, using optimized concentrations of cells and primary mouse anti-*Salmonella* monoclonal antibody (MCA) (Figure 6).

### 2.7. Electrochemical Experimental Procedure

**Figure 7:**
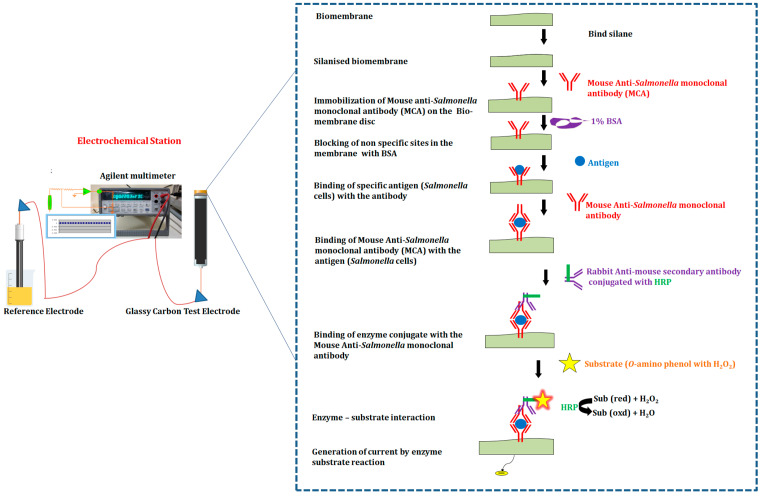
Diagrammatic illustration of immunosensor membrane preparation, operating procedure and detection of *Salmonella* cells.

The following electrochemical experimental procedures were followed. The electrochemical experiments were carried out in a dry sample cell, keeping cadmium as the reference electrode and glassy carbon as the test electrode. The electrodes were connected to a multimeter (Agilent 34401 A), which captures the response signal output current generated by the working electrode when a solution was introduced. An electrolyte solution of 0.1 M PBS at pH 7.2 was introduced into the dry sample cell at the start of an experimental run and the response signal output current was recorded for about 15 min. This response served as a control. A solution containing the mouse anti-*Salmonella* monoclonal antibody (MCA) with glutaraldehyde was then introduced into the cell, first washing out the PBS. The response signal output current was then detected for 15 min. During this stage, the monoclonal antibody was immobilized onto the membrane by means of the cross linker, glutaraldehyde. After immobilization, the unbound antibodies were removed by washing the surface of the working electrode five times with 0.1 M PBS at pH 7.2 solution. Then, a solution of 1% BSA (10 mg/mL) was added to block the unbound areas, and the response signal output current readings were recorded from the point of introduction of BSA, up to 15 min. After blocking, the electrode reaction chamber and the sensor surface were washed five times with 0.1 M PBS solution to remove the unbound BSA. Then, the heat killed cells of *Salmonella* antigen were added and incubated for 30 min. The response produced by the binding of *Salmonella* antigen with the antibody was detected for about 15 min, and then the probe surface with captured *Salmonella* antigen was consecutively washed with 0.1 M PBS and dipped into an electrode reaction chamber containing a rabbit anti-mouse secondary antibody conjugated with HRP (1 µL/mL of PBS). The response signal output current detected by the sensor was measured for about 15 min. After 15 min, the sensor surface was rinsed five times with 0.1 M PBS. The substrate (o-amino phenol (2 mg) with 1.4% hydrogen peroxide dissolved in 1 mL of 0.1 M PBS at pH 7.2 was added, to react with HRP. As the interaction between HRP and substrate proceeded, a steady response signal output current was generated which was related to the association rate constant of antibody and antigen. This was detected and recorded using the Agilent software. All electrochemical measurements were carried out, keeping the working electrode as well as the cadmium electrode upside down in a sample cell. The sample volume was 600 µL for all the experimental steps. All operations were carried out at room temperature (25 °C) (Figure 7). 

### 2.8. Statistical Analysis and Graphs

The statistical differences among the data were computed using one way analysis of variance (ANOVA) and the post hoc Tukey test at the *p* < 0.05 significance level. All of the statistical testing and graphs in this manuscript were created using OriginPro-2020. The mean and standard deviation for each data set (n = 5 samples) was calculated. The electrical response signal output current values for the bare electrode versus the collagen membrane, the collagen membrane versus the agarose membrane, the dialysis membrane electrode interface disc versus the bare electrode, the dialysis membrane electrode interface disc versus the gelatin membrane, the dialysis membrane electrode interface disc versus the agarose membrane, and the dialysis membrane electrode interface disc versus the collagen membrane were compared, and statistical significances were determined.

## 3. Results

### 3.1. Electrodes and Electrochemical Measurement Device

Our studies indicate that the two electrode system viz., the glassy carbon working electrode and the cadmium reference electrode (without potentiostat), connected to an electrochemical measurement device, a high performance 6½ digital Agilent 34401A multimeter (Agilent Technologies, Inc., Santa Clara, CA, USA) with Agilent software, is sufficient to detect the response signal output current generated by the enzyme-substrate reaction resulting from the binding of antigen with immobilized antibody on the disposable biomembrane electrode interface surface. The study further demonstrates that the sensor system in the presence of the analyte transduces electrons and conducts an electrical response signal up to 30 nA without applying any potentiostat current to the electrode system. This may be because of the high potential generated by the cadmium reference electrode. Other reference electrodes viz., silver and copper, that were used in the present study, failed to detect any response signal (data not shown). The electrode measures the current output in milliampere (mA). However, for simplicity in the text, we have referred to the output in nanoampere (nA) (1 milliampere equals to 1,000,000 nanoampere). We have retained the use of milliamp (mA) format in the figures. 

### 3.2. Fabrication of Biomembrane Electrode Interface

**Figure 8:**
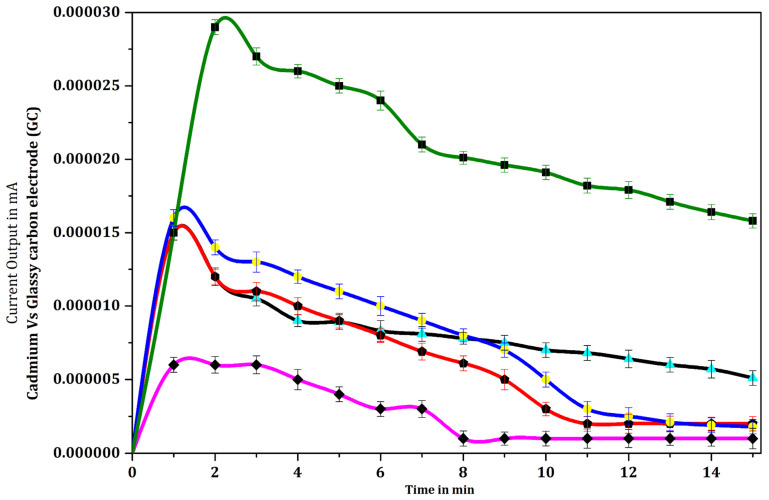
Response signal output current curves of glassy carbon electrode fixed with different membranes. Bare electrode without membrane (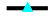), electrode with gelatin membrane (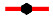), electrode with agarose membrane (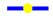), electrode with collagen membrane (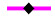) and electrode with a dialysis membrane electrode interface disc (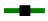). All the membranes were coated with the binder silane, and immobilized with specific mouse anti-*Salmonella* monoclonal antibody (MCA) (1:1000 dilution) followed by specific cells *Salmonella* sp. 3000 cells (3 × 10^5^ cells/mL), and rabbit anti-mouse secondary antibody conjugated with HRP in 0.1 M PBS. Data represent mean ± sd (n = 5).

The dialysis membrane electrode interface disc was found to provide the best electrode interface membrane, giving a more consistent maximum output response signal than gelatin, agarose or collagen. The response signal transduced by the glassy carbon electrode through dialysis membrane electrode interface disc was found to be higher (29 nA), than that generated by the other biomaterials used, namely agarose (18 nA), gelatin (16 nA) and collagen (7 nA). The response signal was only 16 nA from the bare glassy carbon electrode coated with the binder, silane. The gelatin and agarose membranes exhibited the highest output response signal initially but thereafter decreased progressively. Moreover, the response signal outputs recorded from gelatin and agarose membranes were closely similar to those from the bare electrode used in the control experiment. Initially, the dialysis membrane electrode interface disc exhibited a relatively low response signal output (15 nA) compared to that of the gelatin and agarose membranes, but this slowly increased, reaching a maximum within 2 min, and thereafter the response signal output current remained steady over a long period of time. The collagen membrane showed the lowest output response signal amongst the membranes tested here (Figure 8). The diameter of the dialysis membrane interface disc on the electrode was 6 mm, which allowed retention of a maximum of 10 µL of sample. 

Statistical one-way analysis of variance (ANOVA) was computed for pairs of data (Figure 8, Table 1). The following pairs of data showed significant difference (*p* < 0.05): bare electrode vs. collagen membrane; collagen membrane vs. agarose membrane; dialysis membrane electrode interface disc vs. bare electrode; dialysis membrane electrode interface disc vs. gelatin membrane; dialysis membrane electrode interface disc vs. agarose membrane and dialysis membrane electrode interface disc vs. collagen membrane. “*p*” values greater than 0.05, depicting lack of significant difference, were computed for the following pairs of data: bare electrode vs. gelatin membrane; agarose membrane vs. bare electrode; agarose membrane vs. gelatin membrane and collagen membrane vs. gelatin membrane. 

### 3.3. Response Curves for the Immuno-Enzyme-Substrate Reaction

**Figure 9:**
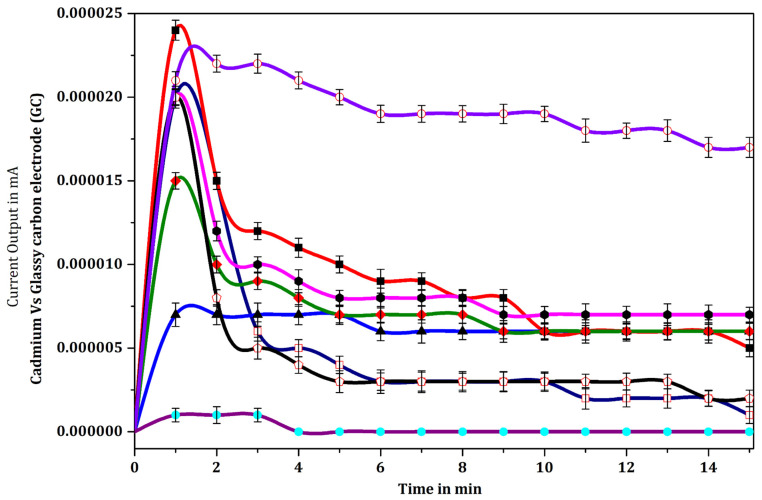
Response signal output current for, respectively, the glassy carbon electrode carrying dialysis membrane electrode interface disc (DM) with phosphate buffer, PBS (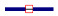); specific mouse anti-*Salmonella* monoclonal antibody (MCA), 1:1000 dilution, immobilized on the electrode (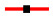); the electrode with DM and MCA with 1% bovine serum albumin, BSA (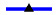); electrode containing DM coated with buffer, MCA and BSA and layered with 3000 *Salmonella* cells (10 µL of 3 × 10^5^ cells/mL), MCA-S.Ag (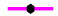); the processed electrode further treated with mouse anti-*Salmonella* monoclonal antibody (1:1000 dilution), MCA-S.Ag-S.mAb (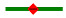); rabbit anti-mouse secondary antibody conjugated with HRP (MCA-S.Ag-S.mAb-HRP), layered on the mouse anti-*Salmonella* monoclonal antibody coated electrode (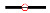); and finally, o-aminophenol substrate coated on the fabricated electrode, MCA-S.Ag-S.mAb-HRP-oAp, in 0.1 M PBS solution (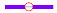). The reactive substrate alone (o-AP in 0.1 M PBS solution) was tested for comparison (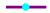). Data represent mean ± SD (n = 5).

Specific binding of *Salmonella* antigen with mouse anti-*Salmonella* monoclonal antibody (MCA) is reflected by the reaction between the bound antibody-enzyme conjugate (rabbit anti-mouse secondary antibody conjugated with HRP) and the substrate o-aminophenol. A 15 min incubation period was found to be sufficient for the detection of enzyme-substrate reaction. In control experiments, the response signals generated by PBS alone or with the addition of 1% BSA, by immobilized specific mouse anti-*Salmonella* monoclonal antibody (MCA) (1:1000 dilution), by 3000 *Salmonella* cells (10 µL of 3 × 10^5^ cells/mL), and by the substrate o-aminophenol alone were similar to one another, with minor variations, and were relatively insignificant. Further, the size of the response signal output current generated in each of the control reactions showed minor variation from that generated with PBS, whereas the enzyme-substrate reaction showed a dramatic increase in the current level, producing about 23 nA in the second minute of the reaction, and maintaining a steady state of 17 nA over a long time period (Figure 9). Though the dialysis membrane electrode interface disc overlayed with mouse anti-*Salmonella* monoclonal antibody (MCA) alone produced a response signal to the level of 23 nA in 60 s, it immediately dropped to 15 nA at the end of 120 s, and thereafter declined until the end of the reaction. Statistical one-way analysis of variance (ANOVA) was computed for pairs of data (Figure 9; Table 2). The following pairs of data showed significant difference (*p* < 0.05): O-aminophenol substrate vs. PBS, primary antibody, BSA, 3000 *Salmonella* cells (3 × 10^5^ cells/mL), secondary antibody, and rabbit anti-mouse secondary antibody conjugated with HRP with substrate; substrate alone vs. PBS, primary antibody, BSA, 3000 *Salmonella* cells (3 × 10^5^ cells/mL), secondary antibody (rabbit anti-mouse secondary antibody conjugated with HRP vs. primary antibody). “*p*” values greater than 0.05, depicting lack of significant difference, were computed for the following pairs of data: BSA vs. PBS, primary antibody, 3000 *Salmonella* cells (3 × 10^5^ cells/mL); primary antibody vs. PBS; 3000 *Salmonella* cells (3 × 10^5^ cells/mL) vs. PBS, primary antibody; rabbit anti-mouse secondary antibody conjugated with HRP vs. PBS, primary antibody, BSA, 3000 *Salmonella* cells (3 × 10^5^ cells/mL); rabbit anti-mouse secondary antibody conjugated with HRP vs. PBS, BSA, 3000 *Salmonella* cells (3 × 10^5^ cells/mL), secondary antibody, substrate alone.

### 3.4. Optimization of the Primary Monoclonal Antibody Concentration

**Figure 10:**
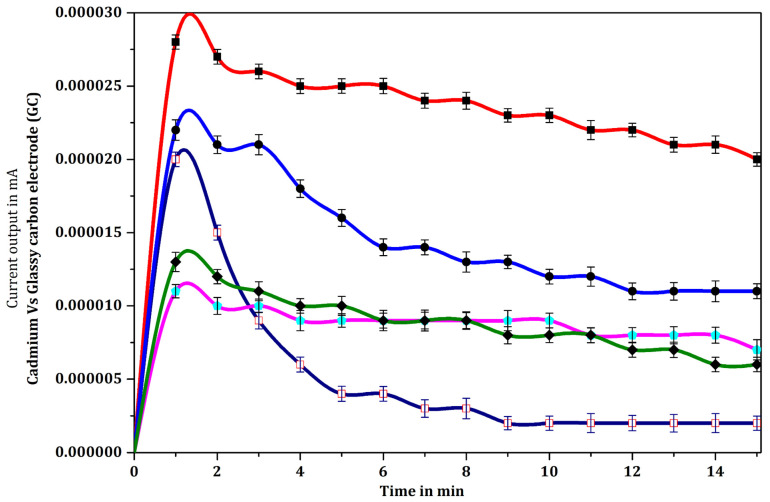
Response signal output current curves for the glassy carbon electrode with PBS (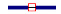), and with various dilutions of immobilized mouse anti-*Salmonella* monoclonal antibody (MCA) on the glassy carbon electrode viz. 1:30,000 (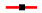), 1:40,000 (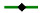), 1:50,000 (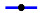), and 1:60,000 (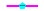). In each case the electrode was prepared with a constant attached population of specific *Salmonella* cells (MCA–S.Ag 3 × 10^5^ cells/mL) and enzyme (HRP) substrate (o-amino phenol) complex in 0.1 M PBS pH 7.2. Data represent mean ± SD (n = 5).

As the response signal of the sensor for the *Salmonella* antigen is dependent on the concentration of the mouse anti-*Salmonella* monoclonal antibody (MCA) immobilized on the disposable biomembrane electrode interface disc, the concentration of the immobilized primary antibody (mouse anti-*Salmonella* monoclonal antibody (MCA)) was optimized, keeping the concentration of *Salmonella* cells constant. The response signal output current from the immunosensor varied depending on the concentration of immobilized primary *Salmonella* antibodies, and the relationship between the response signal output current and the *Salmonella* antibody concentration employed is depicted in Figure 10. For each of the concentrations of primary antibody used (1:30,000, 1:40,000, 1:40,000, 1:60,000), the response signal output current from the immunosensor peaked rapidly within 2 min after the start of the reaction, and thereafter declined at a rate that depended on the antibody concentration. In the case of the 1:30,000 dilution, a peak of 30 nA was achieved, and thereafter the current slowly declined at a relatively uniform rate, reaching 20 nA 15 min after the commencement of the reaction. For the 1:50,000 dilution, the initial peak was lower (23 nA), with an initial steep decline thereafter, reaching a low plateau of 12 nA approximately 12 min after the start of the reaction. With antibody dilutions of 1:40,000 and 1:60,000 a lower peak current was achieved (13 nA and 11 nA, respectively), with a relatively rapid decline to approximately 9 nA within 4–6 min of the start of the reaction, reducing further to approximately 7 nA at 15 min after the start of the reaction, much lower than the response signal output current maintained with the 1:30,000 dilution. Statistical one-way analysis of variance (ANOVA) was computed for pairs of data (Figure 10, Table 3). The following pairs of data showed significant difference (*p* < 0.05): mouse anti-*Salmonella* monoclonal antibody (MCA) in the dilution of 1:30,000 vs. PBS, a monoclonal antibody in the dilution of 1:40,000, 1:50,000, 1:60,000; monoclonal antibody in the dilution of 1:50,000 vs. PBS, monoclonal antibody in the dilution of 1:40,000 and 1:60,000. “*p*” values greater than 0.05, depicting lack of significant difference, were computed for the following pairs of data: PBS vs. mouse anti-*Salmonella* monoclonal antibody (MCA) in the dilution of 1:40,000 and 1:60,000; mouse anti-*Salmonella* monoclonal antibody (MCA) in a dilution of 1:40,000 vs. 1:60,000 (Table 3).

### 3.5. Optimization of Specific Salmonella Antigen Concentration

**Figure 11:**
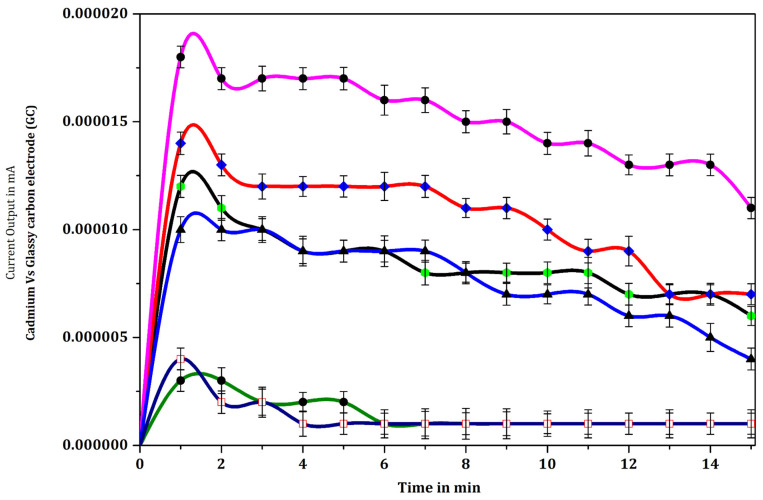
Response signal output current curves of glassy carbon electrode with phosphate buffer solution (PBS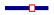) and immobilized mouse anti-*Salmonella* monoclonal antibody (MCA) (1:30,000 dilution) with various concentrations of *Salmonella* cells [MCA-S.Ag 3 × 10^3^ cells/mL (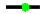), 3 × 10^4^ cells/mL (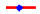), 3 × 10^5^ cells/mL (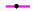), 3 × 10^6^ cells/mL (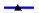), and 3 × 10^7^ cells/mL (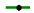)] with MCA-S.Ag-S.mAb-HRP-O-Ap reaction in 0.1 M PBS solution. Data represent mean ± SD (n = 5).

A range of response signal output current was achieved by varying the concentration of *Salmonella* cells (antigen). The electrical current transduction rapidly rose to a maximum level of 19 nA at 60 s after the start of the reaction, and then decreased gradually to the level of 13 nA after an incubation time of 15 min for 3000 cells (10 µL of a 3 × 10^5^ cells/mL suspension of *Salmonella*). In contrast, the response signal output current detected for other concentrations of *Salmonella* cells viz., 30 cells (10 µL of 3 × 10^3^ cells/mL), 300 cells (10 µL of 3 × 10^4^ cells/mL, 30,000 cells (10 µL of 3 × 10^6^ cells/mL and 300,000 cells (10 µL of 3 × 10^7^ cells/mL) were 10.5 nA, 12 nA, 10 nA and 6 nA, respectively, after 120 s of the reaction. The detected response signal output current increased with increasing *Salmonella* cell concentrations in the range 3 cells (10 µL of 3 × 10^2^ cells/mL), 30 cells (10 µL of 3 × 10^3^ cells/mL), 300 cells (10 µL of 3 × 10^4^ cells/mL), and 3000 cells (10 µL of 3 × 10^5^ cells/mL), (Figure 11). In contrast, the detected response signal output current for a concentration of 3 × 10⁶ cells/mL was less than that for 3 × 10^3^ cells/mL, while the signal for 3 × 10⁷ cells/mL was very low and comparable with the response for phosphate buffer solution. The output response signal output current was found to decrease as the bacterial concentration increased above 3000 cells (3 × 10^5^ cells/mL), which was an unexpected finding, possibly indicating the presence of limiting factors such as sensor surface area, antibody immobilization, or antigenic cell capture even under controlled conditions. At higher *Salmonella* cell concentrations the anomaly could be due to a lack of optimized immobilized antibody. The lowest detection limit of response signal output current was determined to be 3 *Salmonella* cells/mL (10 µL of 3 × 10^2^), however the current produced by 3 cells was minimal (0.12 nA) (data not shown). Hence, the optimum level of 30 cells (10 µL of 3 × 10^3^) was used for the detection in our further experiments (Figure 11). Statistical one-way analysis of variance (ANOVA) was computed for pairs of data (Table 4). The following pairs of data showed significant difference (*p* < 0.05): 3000 *Salmonella* cells in the dilution of 3 × 10^5^ cells/mL vs. dilutions of 30 cells/10 µL (3 × 10^3^ cells/mL), 300 cells/10 µL (3 × 10^4^ cells/mL), 30,000 cells/10 µL (3 × 10^6^ cells/mL) and PBS; 300,000 cells/10 µL (3 × 10^7^ cells/mL) vs. dilutions of 30 cells/10 µL (3 × 10^3^ cells/mL), 300 cells/10 µL (3 × 10^4^ cells/mL), 3000 cells/10 µL (3 × 10^5^ cells/mL) and 30,000 cells/10 µL (3 × 10^6^ cells/mL); PBS vs. 30 cells/10 µL (3 × 10^3^ cells/mL), 300 cells/10 µL (3 × 10^4^ cells/mL) and 30,000 cells/10 µL (3 × 10^6^ cells/mL). “*p*” values greater than 0.05, depicting lack of significant difference, were computed for the following pairs of data: 30/10 µL cells/(3 × 10^3^ cells/mL) vs. 300 cells/10 µL (3 × 10^4^ cells/mL), 30,000 cells/10 µL (3 × 10^6^ cells/mL); 30,000 cells/10 µL (3 × 10^6^ cells/mL) vs. 300 cells/10 µL (3 × 10^4^ cells/mL); 300,000 cells/10 µL (3 × 10^7^ cells/mL) vs. PBS.

### 3.6. Determination of the Specificity of the Immunosensor

**Figure 12:**
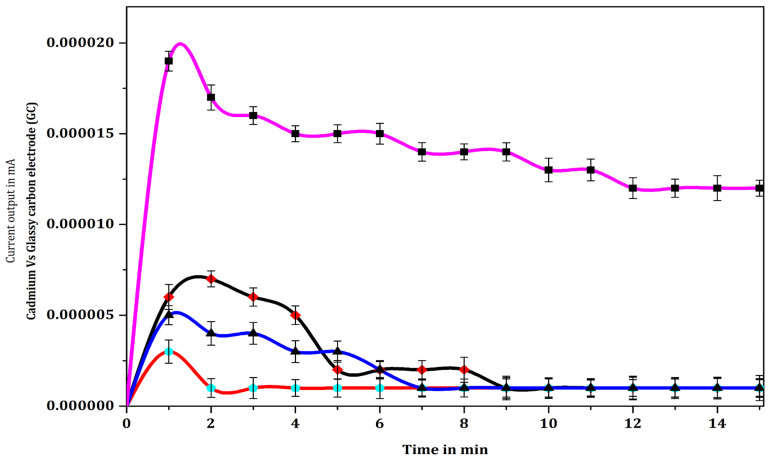
Response signal output current curves derived from the glassy carbon electrode with immobilized specific mouse anti-*Salmonella* monoclonal antibody (MCA) (1:30,000 dilution) in the presence of 30 specific cells of *Salmonella* sp. (10 µL of 3 × 10^3^ cells/mL, 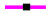) and non-specific cells such as 30 cells of *Vibrio* sp. (3 × 10^3^ cells/mL, 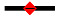), 30 cells of *Pseudomonas* sp. (3 × 10^3^ cells/mL, 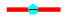) and 30 cells of *Streptococcus* sp. (3 × 10^3^ cells/mL, 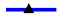) with enzyme and substrate complex. Data represent mean ± SD (n = 5).

The response signal output current for nonspecific cells was compared to that for specific cells (*Salmonella*) using the sensor with optimized antibody and optimized cell concentration. Figure 12 illustrates the response signal output current profile for both specific and non-specific cells. In the case of specific cells *(Salmonella),* having an antigenic epitope complementary to the immobilized mouse anti-*Salmonella* monoclonal antibody (MCA), the best response signal output current was obtained, due to the binding of antigen to the specific antibodies on the sensor surface. In contrast, non-specific bacterial cells (*Vibrio* sp., *Pseudomonas* sp. and *Streptoccocus* sp.), which lacked specific epitope complementary to the immobilized specific mouse anti-*Salmonella* monoclonal antibody (MCA), could not bind to the sensor surface and hence the response signal output current was found to be significantly lower. The response signal output current generated by the sensor for specific *Salmonella* cells was 19 nA after 1 min of reaction, gradually reducing to 12 nA after 15 min. For non-specific cells, *Vibrio*, *Streptococcus* and *Pseudomonas,* the response peaked at 7 nA, 5 nA and 3 nA, respectively, decreasing to only 2 nA within 10 min of reaction, similar to the baseline for the PBS control. The reduced response signal found when cells of *Vibrio* sp., *Pseudomonas* sp., and *Streptococcus* sp. were used, as compared with the response when the same number of *Salmonella* cells was used, implies that the sensor surface with immobilized mouse anti-*Salmonella* monoclonal antibody (MCA) is specific for binding of *Salmonella* cells and will not permit non-selective binding of nonspecific bacterial cells that do not possess epitopes complementary to the immobilized antibody (Figure 12). The results clearly show that a specific response signal is generated in the presence of more than 30 *Salmonella* cells, which represents an improvement in sensitivity over existing sensors currently on the market [4,45,46,47,48,49,50]. Statistical one-way analysis of variance (ANOVA) was computed for pairs of data. The following pairs of data showed significant difference (*p* < 0.05): *Salmonella* sp. vs. *Vibrio* sp., *Pseudomonas* sp., *Streptococcus* sp. “*p*” values greater than 0.05, depicting lack of significant difference, were computed for the following pairs of data: *Vibrio* sp. vs. *Pseudomonas* sp., *Streptococcus* sp.; *Pseudomonas* sp. vs. *Streptococcus* sp. (Table 5).

## 4. Discussion

### 4.1. Electrodes and Electrochemical Measurement Device

A novel technique for identifying antigen-antibody interaction via enzyme-substrate chemical reactions using a two electrode systems without a potentiostat was successfully developed and used, with a simplified experimental methodology. Electrochemical biosensors with three electrodes have already been shown capable of converting a chemical signal into an electrical signal [16,17]. In principle, secondary antibodies conjugated with enzymes bind to antigenic proteins already immobilized on the transducer in biosensors, and as a result of the interaction of the enzyme with the specific provided substrate, the chemical signal is converted into an electric signal, which can be measured using sensors [4,18]. In the two electrode systems described here, cadmium was used as the reference electrode, as opposed to the Ag/AgCI and Calomel electrodes used by previous workers [16,17]. The system detected a maximum electrical signal of 30 nA, despite the fact that there was no known input of applied potential to the electrode system. In the presence of an analyte, the cadmium electrode generates a high potential, allowing it to directly detect antigen-antibody interactions via an enzyme-substrate chemical reaction. The glassy carbon immunosensor system used in the present study was easily applied for monitoring and detecting *S. typhimurium*. The system has higher detection limits than other sensors, yet it only takes 120 s to analyze an enzyme-substrate reaction. The findings from the current study demonstrate the feasibility and consistency of the two-electrode biosensor system for the sensitive detection of *S. typhimurium*. Its ability to provide a wide analytical range, low non-specific binding, steady-state output, and good reproducibility, makes it a useful tool for a wide range of biological applications. The dialysis membrane electrode interface discs can be produced easily and made available until the necessary equipment can be manufactured and tested on a large scale.

### 4.2. Selection of Biomembrane

The current study demonstrated that immobilization of immunoglobulin biomolecules on a disposable biomembrane electrode interface disc bound to the biosensor with silane generates a more reproducible response signal output current than is the case when the sensor is used without a biomembrane electrode interface disc. Howe and Harding [51], on the other hand, reported that antibodies immobilized on the bare sensor surface with silane binder present produced consistent results. As direct immobilization of the primary monoclonal antibody [15] on silanized glassy carbon electrodes without biomatrix was found to generate a lower response signal output current than glassy carbon electrodes with silanized disposable biomembrane interface discs, the current study compared the response signal output current transduced by four different silanized biomembrane electrode interface discs (dialysis, agarose, gelatin, and collagen). The findings from the study clearly show that the dialysis membrane interface disc is a suitable matrix for the glassy carbon electrode, providing for the possibility of testing a large number of samples and automating the technique. It also produces consistent results, in terms of response signal output current, throughout the analysis. The response signal from the glassy carbon electrode using dialysis membrane interface disc with silane binder was the best of the options tested, likely because the pore size and homogeneity offered by the membrane for the binding of primary monoclonal antibody provided the correct orientation for the immobilized antibody to bind with the active site of the *Salmonella* antigen. In contrast, the response signal output current for biomolecules immobilized on agarose, gelatin, and collagen matrices decreased significantly over the time of reaction. This may have been due to the fact that the primary monoclonal antibody molecules were less securely bound to the biomembrane and varied in distribution, due to larger pore size and poorer membrane homogeneity. This resulted in a lower response signal and rapid deterioration of the response signal output current [52]. 

### 4.3. Determination of Immuno-Enzyme Substrate Reaction

Uttenthaler et al., 2001 [53], carried out coupling of antibodies to the biomembrane using a cross-linking reagent. In the current study, it was found that immobilizing monoclonal antibodies onto a dialysis membrane electrode interface disc sensor surface using silane binder and a crosslinker, glutaraldehyde, subsequently facilitated a strong specific binding of antigens and thus capture of *Salmonella* cells. This in turn helped to ensure that the specific reaction of *Salmonella* monoclonal antibody-HRP-o-aminophenol (OAP) with the captured *Salmonella* cells yielded a consistent reaction and response signal output current. The purpose of incorporation of BSA in the reaction mixture was to neutralize charges on the remaining unbound areas of the biomembranes electrode interface disc, thus reducing non-specific adsorption of molecules and facilitating the specific binding and reaction of *Salmonella* monoclonal antibody-*Salmonella* antigen-HRP-o-aminophenol. BSA occupies superfluous, non-specific binding sites on the biomembrane [44] and the response signal output current for the non-specific binding of BSA was negligible. Similar results were observed by Quinn et al., 1999 [15], using a hydrogel-based biointerface. Successive washing of the biomembrane electrode interface disc after each step of the experiment facilitated the specific binding of antigen-antibody-enzyme-substrate and thus prevented the participation in the reaction of unbound molecules on the sensor surface. 

### 4.4. Optimization of Primary Monoclonal *Salmonella* Antibody Concentration

The study has shown that the 1:30,000 dilution of primary antibody (mouse anti-*Salmonella* monoclonal antibody (MCA)) gave the maximum response signal output current of 30 nA during the first 2 min of the reaction, enabling detection of the target *Salmonella* analyte. The response signal output current is generally related to the amount of substrate and to the concentration of primary antibody bound to the membrane of the sensor surface. The variations observed in the response signal output current with different antibody concentrations enabled the selection of the optimum antibody concentration, which was used for subsequent experiments. The results of this study correlate with those of earlier investigations [15,18,19,44,54,55,56]. The response signal output current triggered by the catalytic immunochemical reaction between the HRP enzyme and the substrate may relate to the enzymatic reaction velocity, according to the Michaelis-Menten equation [44,54,55,56]. This, in turn, is determined by the quantity of *Salmonella* antigenic cells present, as well as the primary monoclonal antibody, coupled with enzyme-attached second antibody linked to the immunosensor membrane.

### 4.5. Optimization of *Salmonella* Antigen Concentration

When the concentration of *Salmonella* cells was varied, the limit of detection of the immunosensor (LOD) ranged from 30 cells (3 × 10^3^ *Salmonella* cells/mL) to 30,000 cells (3 × 10^7^ *Salmonella* cells/mL). The response signal output current was found to be reduced as the bacterial concentration increased (i.e., above 3000 cells [3 × 10⁵ cells/mL]), which was an unexpected result, possibly indicating the presence of limiting factors such as sensor surface area, antibody immobilization, or antigenic cell capture even under controlled conditions. The anomaly could possibly reflect a lack of optimized immobilized antibody at higher *Salmonella* cell concentrations. Olsen et al., 2003 [57], reported that the change in steady-state current may be proportional to the step change in antigen concentration. The silicon sensor detected less than 10^3^ cells of *Neisseria meningitidis* in a 20-min ELISA assay, whereas a 2.5 h enzyme-linked immunosorbent assay using the same antigen and antibody preparations revealed a far less sensitive detection (6 × 10^4^) of cells [58]. Similarly, Braiek et al., 2012 [18], demonstrated that an immunosensor has specificity with a linear relationship at a concentration of *S. aureus* cells (10–10^6^ CFU/mL), though reproducibility was found to be very poor (8%). A wireless magneto-elastic mass-sensitive biosensor was used to detect 1.6 × 10^2^ CFU/mL of *Salmonella* [57,59]. Electroanalytical techniques exhibited very low detection limits for bacterial cells (10^9^ M) with small volumes (1–20 µL) of samples. The detection limit of a bioenzyme (tyrosinase and horseradish peroxidase) electrochemical biosensor for *S. typhimurium* was 1.09 × 10^3^ CFU/mL and the detection time was 2.5 h [60]. Anti-*Salmonella* antibody with a limit of detection (LOD) of 10 CFU/mL and a detection time of 125 min was used to detect *Salmonella typhimurium* lipopolysaccharide (somatic “O” antigen) in food and water samples [61,62]. Electrochemical impedance spectroscopy (EIS) confirmed the detection of lipopolysaccharide at 0.001–0.1 g/mL and the LOD of bacteria at 1 × 10^1^ CFU/mL [62]. The performance of the immunosensor compared to the traditional method for the detection of *Salmonella* sp. at concentrations 10^1^ CFU/mL and 10^3^ CFU/mL is a remarkably interesting result. A polyclonal antibody against the recombinant PagC protein was used to capture *Salmonella* from samples and the pagC antibody IMBs-qPCR method’s efficiency, sensitivity, and specificity were demonstrated for the detection of 30 *Salmonella* cells in less than 10 h [63]. In the current study, it was found that the immunosensor device described not only improved the limit of detection (LOD) to 30 cells (3 × 10^3^ *Salmonella* cells/mL) from 30,000 cells (3 × 10^7^ *Salmonella* cells/mL), but also reduced the quantity of reagents and the time required for detecting *Salmonella* cells to 2 min compared to that required by cultural methods (2 to 5 days).

### 4.6. Specificity of the Immunosensor

In order to show the specificity of the immunosensor, the interaction of the immobilized primary monoclonal *Salmonella* antibody with other nonspecific organisms was studied. The results showed a marked difference in response signal output current for *Salmonella,* as compared to other organisms *(Vibrio* sp., *Pseudomonas* sp. and *Streptococcus* sp.), indicating that bacteria which lacked the antigenic determinant site corresponding in specificity with the immobilized *Salmonella* antibody did not bind to the sensor surface and thus failed to generate a specific response signal output current. The signals generated by nonspecific organisms such as *Vibrio* sp., *Pseudomonas* sp. and *Streptococcus,* sp. were significantly lower and showed a different pattern to that observed with *Salmonella* cells, due to their reduced affinity towards the primary immobilized monoclonal *Salmonella* antibody. The role of BSA was to neutralize charges on the unbound areas of the membrane, other than those areas where primary monoclonal antibody was attached, and thus avoid non-specific binding. Adsorption of the analyte (antigen) was limited to the sites of immobilized primary *Salmonella* monoclonal antibodies on the biomembrane [53]. The specificity of the immuno electrochemical biosensor in the presence of other nonspecific organisms was therefore demonstrated [42,57,64,65]. Multiple research projects [4,59,60,64] have lately focused on detecting *Salmonella* using standard analytical approaches such as an immunosensor. As demonstrated in this investigation, monoclonal antibodies are useful in the specific biosensor detection of *Salmonella*. Polyclonal anti-*Salmonella* antibodies were also used to immobilize on the gold working electrode, revealing *S. typhimurium’s* LOD at 10 CFU/mL with a detection time of 125 min [66]. The current recorded during the *S. typhimurium* detection revealed a linear calibration curve up to 10^5^ CFU/mL [66]. A comparative study of the application of various alternatives for the detection and identification of bacteria, such as SERS-based biosensors, MALDI-TOF, colonies-scattering-based optical biosensors [67,68,69,70,71] and even smart phone-based biosensors, would be interesting and informative. At present, more than 2555 serovars of *Salmonella* have been recorded [2,72]. It was judged that biosensor detection of a variety of *Salmonella* serotypes was impractical in the present study. In *Salmonella* research, including genetic approaches for serovar identification, subspecies serotypes are identified using antigenic formulas that follow the subspecies name [72]. Eventually, further biosensor approaches will be developed and their application in *Salmonella* serovar discrimination and treatment will be validated. The equipment and experimental approaches disclosed in this paper could form a basis for distinction of *Salmonella* strains and serovars. The immunosensor described here has been optimized and validated to detect *Salmonella* very specifically, under various experimental conditions, using PBS as the suspending medium. It is likely that the system would work in other situations (and for other pathogenic bacteria), but it may be necessary to undertake additional studies to determine if other constituents or contaminants in the bacterial suspension (originating, for example, from soil or fecal material) would interfere with the electrochemical detection of *Salmonella* serovars. However, in general, the method appears to yield reproducible results and is well suited for bacterial assay, having the advantages of being simple, inexpensive, highly sensitive, and rapid. 

## 5. Conclusions

A novel method for detecting antigen-antibody interaction via enzyme-substrate chemical reaction, using a two-electrode system without a potentiostat, has been successfully developed and used with a simplified experimental methodology. The glassy carbon immunosensor described not only has better detection limits than other sensors, but also requires only 2 min for enzyme-substrate reaction analysis. The features of this biosensor include consistency and sensitivity for the detection of *Salmonella typhimurium*, providing a large analytical range, low non-specific binding, steady state output and good reproducibility. The device and its method of application have potential use in a large variety of biological applications. The current study has also demonstrated that a silanized dialysis membrane interface disc placed on the glassy carbon electrode surface provides an adequate matrix for specific immobilization of antigens and antibodies, and enables more sensitive detection of *S. typhimurium* that has been possible until now. The biosensor has a very sensitive detection limit, viz., three cells, and there is little non-specific reaction. The dialysis membrane interface disc has a higher maximum response signal output current than gelatin, collagen, or agarose. The manufacturing process for the disposable dialysis membrane electrode interface discs is simple and could be employed for large-scale testing. The glassy carbon immunosensor system developed in this study can be easily applied, not only for monitoring and detecting *S. typhimurium*, but also for automated detection of other potentially pathogenic microorganisms. 

## Figures and Tables

**Table 1:** Comparative analysis among different membranes. *p*-value for each pair of data analyzed using one-way ANOVA. The significance value (0, 1) provided in parenthesis beside the *p*-value denotes the significance of each pair of data. Significance value 1 indicates that the data pairs are significantly different (*p*-value lesser than 0.05). The significance value 0 depicts that the data pairs are not significantly different (*p*-value greater than 0.05).

	Bare Electrode	Gelatin	Agarose	Collagen	Dialysis Membrane Electrode Interface Disc
Bare Electrode	-	0.81662 (0)	0.99821 (0)	0.01714 (1)	0 (1)
Gelatin	0.81662 (0)	-	0.093573 (0)	0.22572 (0)	0 (1)
Agarose	0.99821 (0)	0.093573 (0)	-	0.03897 (1)	0 (1)
Collagen	0.01714 (1)	0.22572 (0)	0.03897 (1)	-	0 (1)
Dialysis membrane electrode interface disc	0 (1)	0 (1)	0 (1)	0 (1)	-

**Table 2:** Comparison of response signal output current recorded during the fabrication of sensor. *p*-value for each pair of data analyzed using one-way ANOVA. The significance value (0, 1) provided in parenthesis beside the *p*-value denotes the significance for each pair of data. Significance value 1 indicates that the data pairs are significantly different (*p*-value lesser than 0.05). The significance value 0 denotes that the data pairs are not significantly different (*p*-value greater than 0.05).

	PBS	Mouse Anti-*Salmonella* Monoclonal Antibody	BSA	*Salmonella* Cells (3 × 10^5^ cells/mL)-Antigen	Mouse Anti-*Salmonella* Monoclonal Antibody	Rabbit Anti-Mouse Secondary Antibody	O-Aminophenol Substrate	Substrate Alone
PBS	-	0.07295 (0)	0.98331 (0)	0.1858 (0)	0.70662 (0)	0.99999 (0)	0 (1)	0.04545 (1)
Mouse Anti-*Salmonella* monoclonal antibody	0.07295 (0)	-	0.47224 (0)	0.99993 (0)	0.90661 (0)	0.03544 (1)	0 (1)	0 (1)
BSA	0.98331 (0)	0.47224 (0)	-	0.73392 (0)	0.99529 (0)	0.93439 (0)	0 (1)	0.00232 (1)
*Salmonella* cells (3 × 10^5^ cells/mL)-antigen	0.1858 (0)	0.99993 (0)	0.73392 (0)	-	0.98744 (0)	0.1017 (0)	0 (1)	0 (1)
Mouse Anti-*Salmonella* monoclonal antibody	0.70662 (0)	0.90661 (0)	0.99529 (0)	0.98744 (0)	-	0.53113 (0)	0 (1)	0 (1)
Rabbit Anti-mouse secondary	0.99999 (0)	0.03544 (1)	0.93439 (0)	0.1017 (0)	0.53113 (0)	-	0 (1)	0.09124 (0)
O-aminophenol substrate	0 (1)	0 (1)	0 (1)	0 (1)	0 (1)	0 (1)	-	0 (1)
Substrate Alone	0.04545 (1)	0 (1)	0.00232 (1)	0 (1)	0 (1)	0.09124 (0)	0 (1)	-

**Table 3:** Optimization of monoclonal anti-bodies in different concentration for sensor application. *p*-value for each pair of data analyzed using one-way ANOVA. The significance value (0, 1) provided in parenthesis beside the *p*-value denotes the significance for each pair of data. Significance value 1 indicates that the data pairs are significantly different (*p*-value lesser than 0.05). The significance value 0 depicts that the data pairs are not significantly different (*p*-value greater than 0.05).

	PBS	MCA 1:30,000	MCA 1:50,000	MCA 1:60,000	MCA 1:40,000
PBS	-	0 (1)	0 (1)	0.02574 (0)	0.02631 (0)
MCA 1:30,000	0 (1)	-	0 (1)	0 (1)	0 (1)
MCA 1:50,000	0 (1)	0 (1)	-	0.01477 (1)	0.01581 (1)
MCA 1:60,000	0.02574 (0)	0 (1)	0.01477 (1)	-	1 (0)
MCA 1:40,000	0.02631 (0)	0 (1)	0.01581 (1)	1 (0)	-

**Table 4:** Optimization of cells in different concentration for sensor application. *p*-value for each pair of data analyzed using one-way ANOVA. The significance value (0, 1) provided in parenthesis beside the *p*-value denotes the significance of each pair of data. The significance value 1 indicates that the data pairs are significantly different (*p*-value lesser than 0.05). The significance value 0 depicts that the data pairs are not significantly different (*p*-value greater than 0.05).

	*Salmonella* Cells (3 × 10^3^ cells/mL)	*Salmonella* Cells (3 × 10^4^ cells/mL)	*Salmonella* Cells (3 × 10^5^ cells/mL)	*Salmonella* Cells (3 × 10^6^ cells/mL)	*Salmonella* Cells (3 × 10^7^ cells/mL)	PBS
*Salmonella* cells (3 × 10^3^ cells/mL)	-	0.35092 (0)	0 (1)	0.98036 (0)	0 (1)	0 (1)
*Salmonella* cells (3 × 10^4^ cells/mL)	0.35092 (0)	-	0 (1)	0.08345 (1)	0 (1)	0 (1)
*Salmonella* cells (3 × 10^5^ cells/mL)	0 (1)	0 (1)	-	0 (1)	0 (1)	0 (1)
*Salmonella* cells (3 × 10^6^ cells/mL)	0.98036 (0)	0.08345 (1)	0 (1)	-	0 (1)	0 (1)
*Salmonella* cells (3 × 10^7^ cells/mL)	0 (1)	0 (1)	0 (1)	0 (1)	-	0.99999 (0)
PBS	0 (1)	0 (1)	0 (1)	0 (1)	0.99999 (0)	-

**Table 5:** Sensitivity analysis of sensor among various test samples. *p*-value for each pair of data analyses using one-way ANOVA. The significance value (0, 1) provided in parenthesis beside the *p*-value denotes the significance for each pair of data. Significance value 1 indicates that the data pairs are significantly different (*p*-value lesser than 0.05). The significance value 0 depicts that the data pairs are not significantly different (*p*-value greater than 0.05).

	Non-Specific *Vibrio* sp.	Non-Specific *Pseudomonas* sp.	Non-Specific *Streptococcus* sp.	Specific *Salmonella* sp.
Non-specific *Vibrio* sp.	-	0.39087 (0)	0.9147 (0)	0 (1)
Non-specific *Pseudomonas* sp.	0.39087 (0)	-	0.78309 (0)	0 (1)
Non-specific *Streptococcus* sp.	0.9147 (0)	0.78309 (0)	-	0 (1)
Specific *Salmonella* sp.	0 (1)	0 (1)	0 (1)	-

## Data Availability

Not applicable.
